# Effect of body mass index trajectory on hypertension among children and adolescents aged 5–18 years: a retrospective cohort study

**DOI:** 10.1080/07853890.2023.2267572

**Published:** 2023-10-16

**Authors:** Lu Wang, Longbing Ren, Yuzhu Wang, Zixiang Ji, Rongyu Zhu, Yingxian Sun, Jue Li, Lijuan Zhang

**Affiliations:** a Clinical Center for Intelligent Rehabilitation Research, Shanghai YangZhi Rehabilitation Hospital (Shanghai Sunshine Rehabilitation Center), Tongji University School of Medicine, Tongji University, Shanghai, China; b Department of Cardiology, The First Hospital of China Medical University, Shenyang, Liaoning, China

**Keywords:** Children and adolescents, blood pressure, hypertension, body mass index trajectory, latent class growth mixture model

## Abstract

**Background:**

Hypertension has become increasingly prevalent in Chinese children and adolescents in recent decades, which affects growth and development of children, leads to cognitive decline and multiple target organ damage. Here, we assessed the impact of different body mass index (BMI) trajectories on the occurrence of hypertension in children and adolescents using a cohort study in Northeast China.

**Materials and methods:**

Children and adolescents aged 5–18 years was extracted for physical examination in Fuxin City, Liaoning Province, China during the 2009–2015 period. A latent category growth mixed model (LCGMM) was used to classify BMI changes and analyze the effect of different BMI trajectories on the risk of occurrence of hypertension in these participants within 5 years.

**Results:**

All participates were divided into five BMI trajectories by LCGMM method: slow increasing group (*n* = 2616, 30.8%), overweight and obesity (OW/OB) group (*n* = 1141, 13.4%), normal decreasing group (*n* = 232, 2.7%), stable normal group (*n* = 4383, 51.6%), and fast-increasing group (*n* = 120, 1.4%). Compared with the stable normal group, the slow increasing group [adjusted odds ratio (AOR): 1.610, 95% confidence interval (CI): 1.304–1.989], the OW/OB group (AOR: 3.172, 95% CI: 2.500–4.023) and the fast-increasing group (AOR: 2.708, 95% CI: 1.445–5.074) all increased the risk of developing hypertension in children and adolescents.

**Conclusion:**

The potential of developing hypertension varies among groups of children aged 5–18 with different BMI trajectories. Children and adolescents in the normal BMI range (the slow growth group) still need to be aware of the change in BMI trajectory to stop or slow down the progression of BP abnormalities.

## KEY MESSAGES

1. Five body mass index trajectories from ages 5–18 years were determined using the LCGMM method, which labeled as the slow increasing group, overweight and obesity (OW/OB) group, normal decreasing group, stable normal group, and fast-increasing group.
2. Different BMI trajectories in children and adolescents aged 5–18 years are differentially associated with the development of hypertension.
3. Increased BMI levels in children and adolescents increase the risk of hypertension. Moreover, even within the normal BMI range, a modest growth might raise the risk of high-normal BP.

## 1. Introduction

Hypertension is a major human health hazard and the leading cause of the global disease burden worldwide [1]. The high prevalence of hypertension is mainly due to a combination of factors such as obesity, lack of physical activity, poor diet structure, genetic factors and environmental pollution [2–7]. Hypertension is no longer limited to adults but has also affected young people in the last few decades. The prevalence of hypertension in children aged 6–19 years is about 4.0% and has trended upward over the past 20 years, with a relative increase of 75–79% from 2000 to 2015, in addition to 9.7% of children being prehypertension [8]. The prevalence of hypertension among children and adolescents in China has exceeded 10%, which means that one out of every 10 children may suffer from hypertension [9]. Children with hypertension, especially adolescents, are at increased risk for early onset of subclinical cardiovascular disease [10,11]. Subclinical hypertension target organ damage in children and adolescents, including left ventricular hypertrophy and pathological vascular changes will occur if hypertension risk factors can not be controlled in time [12]. Prevention and management of hypertension in children and adolescents then should be enhanced, with targeted prevention and development of appropriate strategies during the reversible changes and early stages of cardiovascular injury.

Previous studies have identified overweight and obesity (OW/OB) as an important risk factor for hypertension in children [13–15], but most of these studies have focused on BMI at a single point. BMI levels are constantly changing during childhood and adolescence, and the trajectory of BMI changes in children and adolescents is diverse and inconsistent with relatively normal standards, which may lead to a variety of future health outcomes [16–18], so the effect of a single time point on hypertension is limited. Different BMI trajectories have also been shown to be associated with the development of hypertension, however, current studies have obtained inconsistent trajectory-fitting results, such as two studies that both used data from the China Health and Nutrition Survey (CHNS), but fitted different BMI dynamic changing trajectories: three growth trajectories were fitted, a normal increasing group, a high increasing group and a resolving group, where a high increasing group increases the risk of hypertension in children [19]; four distinct BMI trajectories across childhood were identified: lean-stable increase, medium-marked increase, heavy marked decrease, and heavy marked increase, and it was determined that participants in the heavy markedly increase group were at the highest risk [20]. The differences in these results may be due to differences in social and environmental factors such as lifestyle habits in different regions, resulting in different BMI trajectories for children and adolescents [21–24]. Currently, there are few studies on the effect of BMI trajectories on hypertension in children in China. Considering the differences in BMI in children from different regions and the lack of evidence of continuous observations, regional studies with large sample sizes are still needed to elucidate the effect of BMI on blood pressure.

We used a latent category growth mixed model (LCGMM) to explore several potential types of trajectories of change in BMI and clarify the impact of different trajectories on the occurrence of hypertension in children and adolescents aged 5–18 years using a 5-year cohort in Northeast China. We selected Fuxin city of Liaoning Province as a study district based on the following reasons. Fuxin is one of the sub-centers of the Shenyang Economic Zone and has a medium economic level, which can represent the development level and economic structure of most cities in China; in addition, the population of Fuxin is less mobile and the data is easier to track, which can better represent the typical city situation in China.

## 2 Materials and methods

### 2.1. Participants

**Figure 1.**
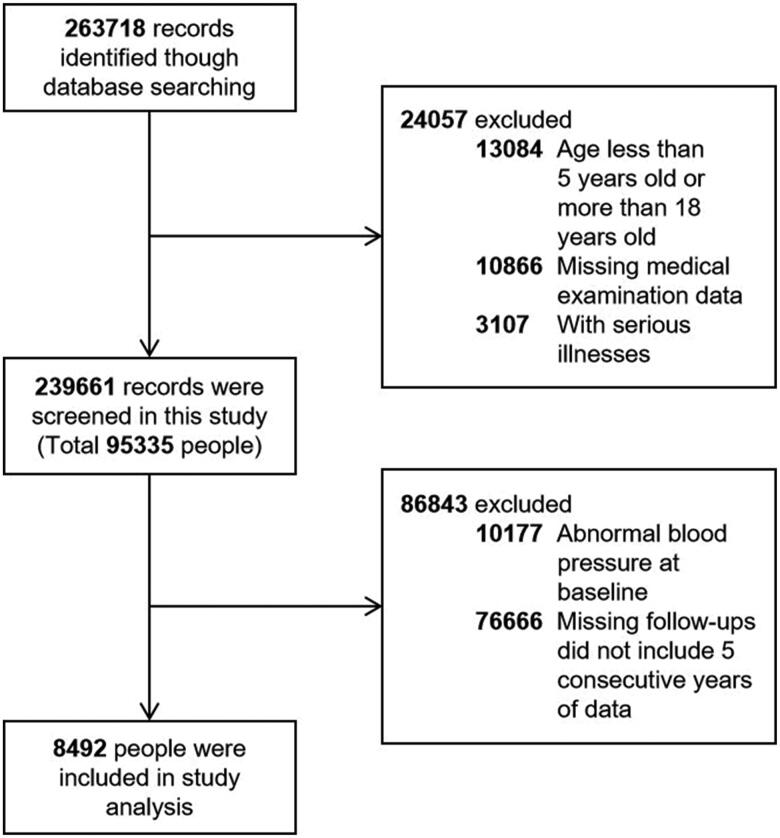
Flow chart for study population.

The data were derived from the annual routine physical examinations database for primary, middle, and high schools in Fuxin City, Liaoning Province, China during 2009–2015. We used the stratified multi-stage sampling method and randomly selected primary or secondary schools from five municipal districts. Students aged 5–18 years old without previous serious illnesses were included in this study and the selection details were presented in Figure 1. Briefly, a total of 263,718 routine physical examination data were obtained by searching the database; 76,666 subjects were missing from the follow-up; a total of 10,177 individuals with abnormal blood pressure at baseline were excluded; finally, 8492 participants with normal blood pressure at baseline and followed up at least 5 years were ultimately screened for the analysis.

### 2.2. Ethics approval and consent to participate

All study procedures were conducted according to the ethical guidelines of the 2000 Declaration of Helsinki. This investigation was approved by the responsible committee of the Ethics Committee of Tongji University, project number 2018yxy08. The requirement of informed consent was waived because the data set used in the present study is anonymous and retrospectively retrieved.

### 2.3. Measurements and definition of BMI/BMIz in children and adolescents

Height and weight were measured using the height-weight scale (RGZ-120) to the nearest 0.1 cm and 0.1 kg, with barefoot, standing straight and hands drooping naturally. Each subject was measured twice and averaged. BMI was calculated as the weight in kilograms divided by the height in meters squared, used to determine OW/OB. For children aged 5 years, the 2006 World Health Organization (WHO) Child Growth Standards [25] is recommended for judgment by China’s child health care technical specification [26] and health industry standards. For 6–18 years old of children, the National Standard of the People’s Republic of China (WS/T 586-2018) was used to classify. Considering the BMI standards of children in different age groups are inconsistent [27], the BMI level of each participant is standardized to generate a new standardized variable. BMI z-score (BMIz) was calculated as (the current BMI level-average BMI level for this sex and age)/standard deviation of BMI by sex and age.

### 2.4. Measurements and definition of blood pressure

An unified instrument was used to measure blood pressure (HEM-7136, Omron). The subject sat, rolled up the right arm sleeve to the armpit, or removed it. Sleeves on the sides. The operator removes the gas from the sphygmomanometer cuff and smoothly binds it to the subject’s upper arm. The cuff should not be too loose or too tight to affect the accuracy of the measurement. When binding the cuff, pay special attention to the cuff’s center. Put the part on the inside of the arm, 1-2 cm above the elbow fossa, and press the humerus with your fingers. The heartbeat can be felt. Participants were advised to relax for 5 min before the assessment and to remain quiet and motionless during the measurement. The right arm’s blood pressure was measured three times, with a 0.5-1 min gap between each test. Systolic and diastolic blood pressure levels were measured. Repeat the measurement if the difference of the results of two adjacent readings is greater than 10 mmHg.

The blood pressure was determined according to the 50th, 90th, 95th, and 99th percentile values corresponding to the levels of sex, age, and height. According to the 2018 Chinese guidelines for the management of hypertension [28], hypertension was defined as systolic BP (SBP) and (or) diastolic BP (DBP) ≥95th percentile, the high-normal BP was defined as SBP and (or) DBP between 90th and 95th percentile or ≥120/80 mmHg.

### 2.5. Statistical analysis

The screening was conducted according to BP at baseline, and children with normal BP entered the model. The latent category growth mixed model (LCGMM) was used to classify the changes in BMIz trajectory [29]; sex and region as covariates were used to adjust the differences between sex and region. The model allows for repeated measurements of variables and different times of measurement records at unequal intervals. The model computes maximum likelihood estimates of growth curve parameters, including fixed-effect parameters and random-effects parameters [19]. We fitted models from 1 class to 5 class of beta, linear, and spline curves, respectively. Model selection was based on the following criteria: Bayesian information criterion (BIC) is closest to 0; high mean posterior class membership probabilities >0.65; high mean posterior probabilities >0.7 [30]. The analysis is carried out using the ‘lcmm’ package in R [31].

All statistical analyses were conducted using R (version 4.0.2). Analysis of two-sample *t*-test, Mann–Whitney test, and Kruskal–Wallis’s test was used for continuous variables, and *χ*^2^ test was used for categorical variables to compare the differential characteristics of different trajectory groups in children and adolescents. After a latent category classification of changes in BMIz trajectories, the data were included in a generalized linear mixed model with school as a random effect, the outcome of hypertension as the dependent variable, and the maximum number of stable normal groups as a reference to assess the effect of different trajectories of BMI change on the risk of developing hypertension in children and on different blood pressure subgroups (DBP/SBP). Age, region and sex were adjusted in the model. *p* < 0.05 to indicate statistical significance.

## 3. Results

### 3.1. Basic information and characteristics among these children and adolescents

Table 1 shows the anthropometric distribution of all study participants by age and sex. A total of 8492 participants aged 5–18 years were included in the analysis, 4101 (48.3%) were boys and 4391 (51.7%) were girls, a relatively adequate sample size for each sex and age group, and the mean BMI of participants at baseline was 17.6 ± 3.4 kg/m^2^, SBP was 104.1 ± 10.5 mmHg, and DBP was 64.3 ± 8.3 mmHg.

**Table 1.** Anthropometric distribution of study population by age and sex.

Age	*N*	Height (cm)	Weight (kg)	BMI (kg/m^2^)	SBP (mmHg)	DBP (mmHg)
Boys						
5 Years	98	121.3 ± 5.8	24.6 ± 5.0	16.6 ± 2.6	99.1 ± 9.0	62.4 ± 7.2
6 Years	967	123.4 ± 5.7	25.9 ± 5.9	16.9 ± 2.9	100.1 ± 9.4	62.1 ± 8.1
7 Years	893	127.9 ± 5.6	28.2 ± 6.5	17.1 ± 3.0	101.9 ± 9.3	63.4 ± 8.2
8 Years	509	133.3 ± 6.0	32.2 ± 8.0	17.9 ± 3.4	104.0 ± 8.7	64.9 ± 7.8
9 Years	427	139.1 ± 6.3	36.0 ± 9.0	18.5 ± 3.5	106.1 ± 9.5	64.9 ± 8.5
10 Years	350	143.8 ± 6.7	39.9 ± 10.2	19.1 ± 3.8	107.7 ± 9.9	65.3 ± 8.3
11 Years	280	149.3 ± 7.8	45.0 ± 13.0	20.0 ± 4.4	109.4 ± 9.7	67.1 ± 7.5
12 Years	217	158.5 ± 8.1	52.6 ± 13.7	20.7 ± 4.1	112.2 ± 9.1	68.0 ± 7.7
13 Years	239	163.7 ± 7.8	56.0 ± 13.0	20.7 ± 3.9	114.5 ± 8.2	68.9 ± 8.4
14 Years	121	168.6 ± 7.2	62.9 ± 14.8	21.9 ± 4.3	115.7 ± 10.0	69.5 ± 7.5
Girls						
5 Years	192	119.2 ± 6.0	22.4 ± 4.3	15.7 ± 2.2	97.8 ± 8.8	61.6 ± 7.4
6 Years	1088	121.3 ± 5.3	23.4 ± 4.3	15.8 ± 2.2	98.1 ± 9.4	61.6 ± 7.9
7 Years	877	126.0 ± 5.7	25.8 ± 5.4	16.2 ± 2.5	101.2 ± 9.5	63.2 ± 8.1
8 Years	482	130.6 ± 6.2	28.5 ± 6.4	16.6 ± 2.9	102.9 ± 9.4	63.8 ± 8.0
9 Years	427	137.4 ± 6.6	32.7 ± 7.6	17.2 ± 3.0	105.2 ± 9.7	64.8 ± 8.4
10 Years	356	143.4 ± 7.3	36.4 ± 8.6	17.5 ± 3.1	107.2 ± 9.2	65.4 ± 8.3
11 Years	287	149.6 ± 7.4	41.2 ± 9.7	18.3 ± 3.3	109.8 ± 9.7	67.0 ± 7.6
12 Years	269	157.1 ± 6.0	47.7 ± 9.2	19.3 ± 3.3	110.9 ± 8.4	67.0 ± 7.9
13 Years	288	158.3 ± 5.5	48.8 ± 9.3	19.4 ± 3.3	112.4 ± 7.9	68.0 ± 7.5
14 Years	125	160.4 ± 5.3	53.2 ± 10.5	20.6 ± 3.3	112.4 ± 8.2	70.2 ± 7.5
Total	8492	135.1 ± 14.8	33.2 ± 12.7	17.6 ± 3.4	104.1 ± 10.5	64.3 ± 8.3

*Abbreviations*: BMI: body mass index; SBP: systolic blood pressure; DBP: diastolic blood pressure.

Anthropometric distribution was described using mean ± standard deviation.

### 3.2. BMI trajectories: the optimal number of BMI trajectories

A total of 917 cases of hypertension occurred in these participants, with a 5-year incidence rate of 10.8%. BMIz values were included in the LCGMM to identify subgroups that followed similar BMIz advances. We fitted models from 1 class to 5 class of beta, linear, and spline curves, respectively. Based on BIC, models with four and five trajectories exhibited significantly better model fits than other models with two and three trajectories. Five fitting methods with the lowest BIC are selected for posterior probabilities comparison (BIC = 52526.25; 52124.62; 52943.34; 52941.11; 51554.19). We observed that model 5 is the most appropriate model with 5 trajectories and a cubic function of age. This model has the higher posterior probabilities and group membership probability and has the smallest BIC value among the 5 models (BIC = 51554.19). The details of the best-fitting model are presented in Tables 2 and 3.

**Table 2.** Model fit statistics (BIC) of different methods for 1–5 latent classes.

Fitting methods	Polynomial degree	Class 1	Class 2	Class 3	Class 4	Class 5
Beta	Linear	58088.87	54894.72	54099.10	54133.12	54234.52
	Quadratic	58078.06	54803.63	57481.06	53349.26	56103.13
	Cubic	58055.63	54781.78	53831.37	54035.84	54099.93
Linear	Linear	65260.89	61447.96	59683.71	58838.77	58184.07
	Quadratic	65262.02	61371.01	59490.31	58365.22	57228.79
	Cubic	65246.87	61357.77	59482.54	58213.42	56931.93
Splines	Linear	56833.68	53683.19	53192.22	**52526.25**	**52124.62**
	Quadratic	56818.14	53589.33	52972.15	53659.20	53066.48
	Cubic	56797.03	53569.18	**52943.34**	**52941.11**	**51554.19**

*Abbreviations*: BIC: the Bayesian information Criterion.

No. Latent class: latent class number of the model.

The five fitting methods with the lowest BIC are highlighted in bold characters.

**Table 3.** LCGMM results of model fitting process.

	BIC	Mean posterior probabilities	Posterior probabilities >0.7 (%)
Model 1	52943.34	0.79; 0.87; 0.77	67.0; 83.2; 64.3
Model 2	52941.11	0.55; 0.62; 0.80; 0.74	9.6; 32.5; 71.6; 58.8
Model 3	52526.25	0.77; 0.76; 0.79; 0.77	64.3; 59.0; 71.3; 63.5
Model 4	52124.62	0.76; 0.74; 0.80; 0.83; 0.81	64.9; 57.8; 63.8; 78.1; 67.8
**Model 5**	**51554.19**	**0.79; 0.79; 0.93; 0.87; 0.79**	**68.5; 66.3; 89.2; 83.0; 67.2**

Abbreviations: BIC, the Bayesian information Criterion; LCGMM, Latent Class Growth Mixture models.

The fitting methods used by the five fitting models were all splines.

Model 1: 3-class cubic latent class growth mixture model; Model 2: 4-class cubic latent class growth mixture model; Model 3: 4-class linear latent class growth mixture model; Model 4: 5-class linear latent class growth mixture model; Model 5: 5-class cubic latent class growth mixture model.

The best fitting model is highlighted in bold characters.

**Figure 2.**
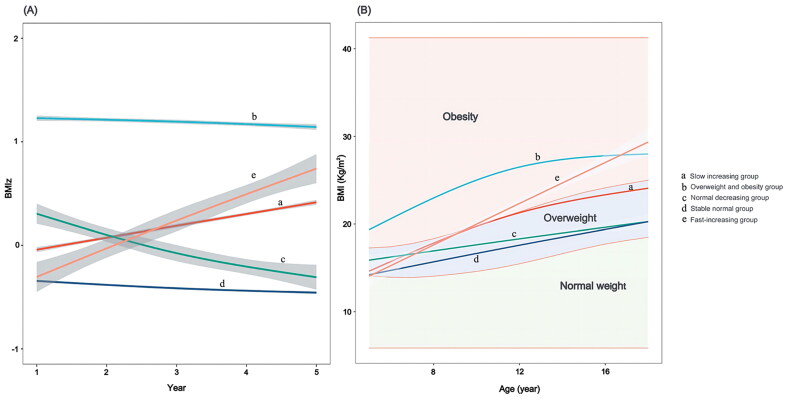
Trajectories of BMI in each group under the best-fit model grouping. (A) BMIz and year of enrollment in each group under the best-fit model grouping; (B) BMI level and age under the best-fit model. Solid lines show the five trajectories estimated from the best-fitting growth mixture model. Shadow shows the 95% confidence intervals. Shaded areas indicate normal (green), overweight (blue), and obese body mass index status (red) across the observed period.

Based on the aforementioned best-fitting model, Figure 2 showed the five diverse trajectories of BMI, in which the BMIz and BMI values were fitted, respectively. The BMI trajectories can be labeled as the slow increasing group (Class 1), OW/OB group (Class 2), normal decreasing group (Class 3), stable normal group (Class 4), and fast-increasing group (Class 5). In the slow increasing group, the BMI levels maintained a small increase (30.8%, *n* = 2616); in the OW/OB group, the BMI levels were stable within the obese or overweight range (13.4%, *n* = 1141); within the normal range, BMI levels declined consistently in the normal decreasing group (2.7%, *n* = 232); children and adolescents in the stable normal group had generally steady BMI trajectories within a normal level (51.6%, *n* = 4383); and the BMI levels in the fast-increasing group rose rapidly within the initial normal range and eventually were OW/OB at age 18 years (1.4%, *n* = 120). Table 4 described the baseline characteristics and hypertension outcome by trajectory groups. Children and adolescents had significant differences (*p* < 0.05) in baseline sex, initial BMI level, BMIz score, and abnormal blood pressure outcomes across the five BMI trajectories.

**Table 4.** Characteristics at baseline and hypertension outcome in children and adolescents by BMI trajectory groups.

	Slow increasing group (*N* = 2616)	Overweight and obese group (*N* = 1141)	Normal decreasing group (*N* = 232)	Stable normal group (*N* = 4383)	Fast-increasing group (*N* = 120)	*p* Value
Age (years)	8.31 ± 2.33	8.41 ± 2.45	8.09 ± 2.39	8.44 ± 2.48	8.35 ± 2.32	0.076
Sex (%)						<0.001
Girls	1388 (53.1)	518 (45.4)	111 (47.8)	2321 (53.0)	53 (44.2)	
Boys	1228 (46.9)	623 (54.6)	121 (52.2)	2062 (47.0)	67 (55.8)	
Region (%)						0.827
Urban	2610 (99.8)	1137 (99.6)	232 (100.0)	4370 (99.7)	120 (100.0)	
Rural	6 (0.2)	4 (0.4)	0 (0.0)	13 (0.3)	0 (0.0)	
BMI (kg/m^2^)	17.31 ± 2.66	23.09 ± 3.40	19.06 ± 3.89	16.27 ± 2.19	15.34 ± 2.72	<0.001
BMIz	−0.10 ± 0.66	1.61 ± 0.82	0.45 ± 1.02	−0.42 ± 0.51	−0.69 ± 0.69	<0.001
hypertension (%)	307 (11.7)	1141 (20.0)	232 (11.2)	335 (7.6)	21 (17.5)	<0.001

*Abbreviations*: BMI: body mass index; BMIz: standardized body mass index; BP: blood pressure.

### 3.3. Association between BMI trajectories with hypertension among children and adolescents

After adjusting for age, region, and sex in the model. Table 5 presented outcomes for the generalized linear regression used to analyze the associations between BMI trajectory groups and the risk of hypertension outcome. Compared with the reference group (stable normal group), the slow increasing group (AOR = 1.610, 95% CI 1.304–1.989), OW/OB group (AOR = 3.172, 95% CI 2.500–4.023), and fast-increasing group (AOR = 2.708, 95% CI 1.445–5.074) increased the risk of hypertension in children and adolescents by about 61, 217, and 171%, respectively (*p* < 0.05).

**Table 5.** The effect of BMI trajectory groups on the risk of hypertension outcome in children and adolescents.

		Reference	*β*	S.E	*p* Value	AOR (95% CI)^a^
BMI trajectory	Slow increasing group	Stable normal group	0.476	0.107	<0.001	1.610 (1.304–1.989)
	Overweight and obese group		1.154	0.121	<0.001	3.172 (2.500–4.023)
	Normal decreasing group		0.452	0.279	0.106	1.572 (0.909–2.718)
	Fast-increasing group		0.996	0.320	0.002	2.708 (1.445–5.074)

*Abbreviations*: BMI: body mass index; AOR: adjusted odds ratio; CI: confidence interval.

^a^Adjust for age, sex, and region.

Table 6 explored distinct hypertension outcomes (SBP and DBP). The findings revealed that, when compared to the stable normal group, all four BMI change trajectories increased the risk of abnormal SBP, but only the OW/OB group increased the risk of abnormal DBP (AOR = 2.363, 95% CI 1.739–3.210), with the difference was significant after adjusting for age, region, and sex (*p* < 0.05).

**Table 6.** The effect of BMI trajectory on abnormal SBP/DBP outcome in children and adolescents using a generalized linear mixed model.

		Reference	*β*	S.E	*p* Value	AOR (95% CI)^a^
Model 1: SBP						
BMI trajectory	Slow increasing group	Stable normal group	0.531	0.144	<0.001	1.701 (1.561–1.853)
	Overweight and obese group		1.203	0.056	<0.001	3.331 (2.984–3.719)
	Normal decreasing group		0.317	0.119	0.008	1.373 (1.086–1.736)
	Fast-increasing group		1.111	0.151	<0.001	3.037 (2.258–4.084)
Model 2: DBP						
BMI trajectory	Slow increasing group	Stable normal group	0.191	0.134	0.154	1.211 (0.931–1.575)
	Overweight and obese group		0.860	0.156	<0.001	2.363 (1.739–3.210)
	Normal decreasing group		0.156	0.363	0.667	1.169 (0.574–2.384)
	Fast-increasing group		0.495	0.451	0.272	1.641 (0.678–3.973)

*Abbreviations*: BMI: body mass index; AOR: adjusted odds ratio; CI: confidence interval; SBP: systolic blood pressure; DBP: diastolic blood pressure.

^a^Adjust for age, sex, and region.

## 4. Discussion

We analyzed physical examination data from 8492 children and adolescents aged 5–18 years in Fuxin, Liaoning Province, China, to identify 5 different BMI trajectories and discuss the effects of different BMI trajectories on the occurrence of hypertension in children and adolescents. This study is one of the first studies to focus on the BMI trajectories of children and adolescents in northern China to give a reference value for the growth trajectory of children and adolescents in northern China.

This study identified five BMI trajectories from ages 5 to 18 years. The majority (51.6%) were in the stable normal group, in which BMI stayed steady within normal weight limits. 30.8% of their BMI levels increased slowly within the normal range, and 13.4% of their BMI levels were stable within the range of OW/OB, 2.7% of the participant’s BMI levels decreased relatively steadily within the normal range, while 1.4% of their BMI levels increased rapidly within the initial normal range and eventually became overweight and obese at the age of 18. High BMI levels and slow increases or decreases in the normal range can increase the risk of hypertension. However, very few studies have emerged on BMI trajectories in children and adolescents and different trajectory-fitting results. Wang et al. [32] fitted four BMI trajectories among children aged 6–18 years in Guangdong Province and found that children with high BMI trajectories had a higher risk of hypertension, all trajectories in this study did not intersect with others and were related to the initial value, which was different from our trajectory fitting. Ji et al. [19] used 1907 children and adolescents aged 6–18 years in the data of the China Health and nutrition survey to fit three BMI growth trajectories: normal increasing group, high increasing group, and resolving group, among which the high increasing group of BMI will increase the risk of hypertension in children. This is consistent with our research that during the growth of children and adolescents, the BMI of some is not stable, and may fluctuate relatively by hormones or hormone levels, personal subjective will, and environment. Buscot et al. [29] studied a cohort of 2631 Finns aged 6–49 years and found that 55.2% of them had a consistent normal BMI level between the ages of 6 and 18 and that this level remained stable into adulthood. About 33.4% of children and adolescents’ BMI levels increased within the normal range in childhood and reached the level of overweight or obesity after middle age, 4.2 and 1.2% of them remained OW/OB under the age of 18 and continued into adulthood, 1.6% of children and adolescents’ BMI levels gradually decreased, while 4.3% of that rose rapidly, the proportion of fitting trajectories in each group is similar with the current study.

Furthermore, studies have demonstrated that elevated BMI levels in childhood and adolescence can irrevocably alter arterial anatomy, resulting in changes in artery-related markers and the risk of atherosclerosis in maturity and that this change remains irreversible even after weight loss [33], which is in line with our findings. We clarified that not only a fast-increasing but a slow-increasing in BMI levels within the normal BMI range in children and adolescents could increase the risk of hypertension. The reason might be that cardiovascular risk factors increase with the change of BMI, even the BMI increasing slowly [34–36]. Additional high hypertension risk at any level of weight gain, per 1-unit change in the distance from the median BMI increases 1.04 (95%CI, 1.04–1.05) times of hypertension risk for youths [34]. Regular physical examinations of children and adolescents should be performed and BMI trajectories should be determined. Close monitoring and timely intervention for targeted prevention are essential for children and adolescents who tend to elevate their BMI in childhood or are at higher BMI levels.

This study had several strengths. Compared with previous studies, the sample size of this study is relatively larger, and the included data are continuous annual follow-ups in a single city. Therefore, the growth status of children and adolescents may be less affected by region, diet, living habits, and other factors, resulting in more accurate fitted trajectories. In addition, we used various methods to fit the trajectory, and the fitting results are more in line with the actual situation than in previous studies. Meanwhile, we discovered that sluggish BMI growth within the normal range increases the risk of high-normal BP in Chinese children and adolescents. Other studies of Chinese children and teenagers have not found this.

There are also some limitations to consider: firstly, this study lacks data on obesity-related indicators such as fat distribution and subcutaneous fat, as well as classifying the level of OW/OB based on BMI only; secondly, it is recommended that children have at least three separate blood pressure values measured to assess hypertension, the blood pressure in this study was a single day with multiple measurements, which still needs to be validated by more accurate clinical trials considering the fluctuation of blood pressure and white coat hypertension (WCH); in addition, our study only involved a population of children and adolescents in Fuxin City, Liaoning Province, China, and although it is representative of some characteristics and conditions in most cities in China, it still has its geographical and limitations, and further data from other cities are needed for subsequent comparative validation.

## 5. Conclusions

In conclusion, our results suggest that the BMI trajectories of different children and adolescents are diverse. Excessive BMI levels and slowly increasing or decreasing in the normal range will increase the risk of hypertension. Children and adolescents within the normal BMI range still need to be aware of the change of BMI trajectory to prevent the development of blood pressure abnormalities. As a result, preventing OW/OB in children and adolescents is critical.

## Data Availability

The data that support the findings of this study are available from the corresponding author LZ upon reasonable request.
